# Recent Reticulate Evolution in the Ecologically Dominant Lineage of Coccolithophores

**DOI:** 10.3389/fmicb.2016.00784

**Published:** 2016-05-24

**Authors:** El Mahdi Bendif, Ian Probert, Francisco Díaz-Rosas, Daniela Thomas, Ger van den Engh, Jeremy R. Young, Peter von Dassow

**Affiliations:** ^1^Marine Biological Association of the UKPlymouth, UK; ^2^Université Pierre et Marie Curie (Paris VI), Roscoff Culture Collection, Station Biologique de RoscoffRoscoff, France; ^3^Centre National de la Recherche Scientifique, FR2424, Station Biologique de RoscoffRoscoff, France; ^4^Facultad de Ciencias Biológicas, Pontificia Universidad Católica de ChileSantiago, Chile; ^5^Instituto Milenio de OceanografíaConcepcion, Chile; ^6^UMI 3614, Evolutionary Biology and Ecology of Algae, Centre National de la Recherche Scientifique-UPMC Sorbonne Universités, PUCCh, UACH, Station Biologique de RoscoffRoscoff, France; ^7^Center for Marine CytometryConcrete, WA, USA; ^8^Departments of Earth Sciences, University College LondonLondon, UK

**Keywords:** coccolithophores, cyto-nuclear discordance, *Emiliania*, evolution, *Gephyrocapsa*, introgressive hybridization, diversity, *Reticulofenestra*

## Abstract

The coccolithophore family Noëlaerhabdaceae contains a number of taxa that are very abundant in modern oceans, including the cosmopolitan bloom-forming *Emiliania huxleyi*. Introgressive hybridization has been suggested to account for incongruences between nuclear, mitochondrial and plastidial phylogenies of morphospecies within this lineage, but the number of species cultured to date remains rather limited. Here, we present the characterization of 5 new Noëlaerhabdaceae culture strains isolated from samples collected in the south-east Pacific Ocean. These were analyzed morphologically using scanning electron microscopy and phylogenetically by sequencing 5 marker genes (nuclear *18S* and *28S rDNA*, plastidial *tufA*, and mitochondrial *cox1* and *cox3* genes). Morphologically, one of these strains corresponded to *Gephyrocapsa ericsonii* and the four others to *Reticulofenestra parvula*. Ribosomal gene sequences were near identical between these new strains, but divergent from *G. oceanica, G. muellerae*, and *E. huxleyi*. In contrast to the clear distinction in ribosomal phylogenies, sequences from other genomic compartments clustered with those of *E. huxleyi* strains with which they share an ecological range (i.e., warm temperate to tropical waters). These data provide strong support for the hypothesis of past (and potentially ongoing) introgressive hybridization within this ecologically important lineage and for the transfer of *R. parvula* to *Gephyrocapsa*. These results have important implications for understanding the role of hybridization in speciation in vast ocean meta-populations of phytoplankton.

## Introduction

**Figure 1:**
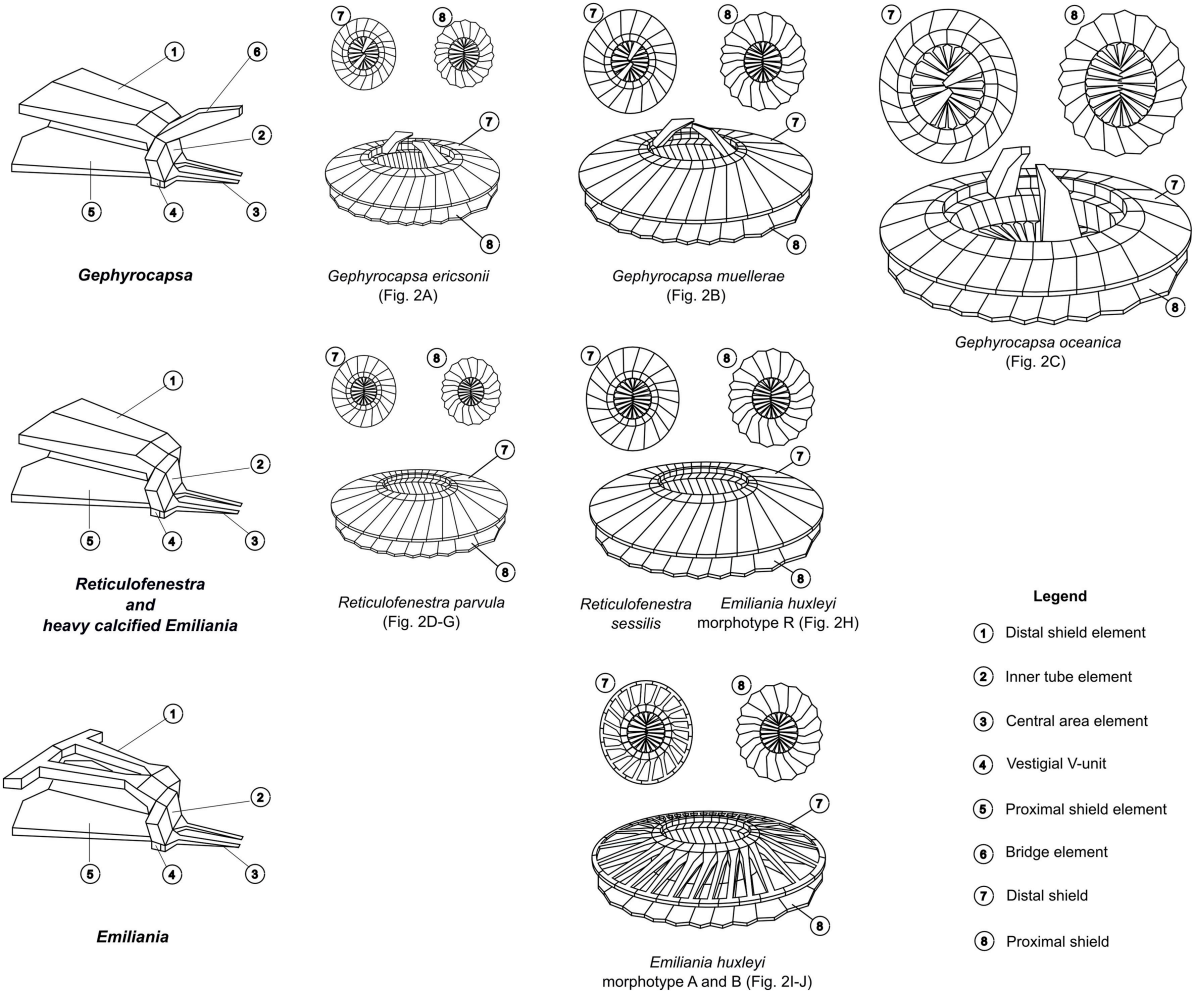
**Coccolith structure of representative Noëlaerhabdaceae redrawn from Young et al. (1992) and Bendif et al. (2014)**. Each morphospecies is associated to its respective SEM image in Figure 2.

Members of the coccolithophore family Noëlaerhabdaceae have numerically dominated coccolithophore communities over the last 20 million years and continue to do so in present day oceans (Raffi et al., 2006). All noëlaerhabdaceaens exhibit the same basic heterococcolith structure that is distinctive among coccolithophores in that crystal V-units are vestigial while R-units form both shields, the two-layered central tube, the central area grill and any central area structures (Young et al., 1992, 2004; Hoffmann et al., 2014; a schematic explaining key aspects of noëlaerhabdaceaen coccolith structure and its variability is provided in Figure 1). The Noëlaerhabdaceae dominated most Neogene nannofossil assemblages with the dominant genera being successively *Cyclicargolithus* Bukry (NN1-6), *Reticulofenestra* Hay, Mohler and Wade (NN6-16), *Pseudoemiliania* Gartner (NN16-19), *Gephyrocapsa* Kamptner (NN19-20), and *Emiliania* Hay and Mohler (NN21) (Bown, 1998). Large-scale blooms of the two most prominent modern coccolithophores, *Emiliania huxleyi* Lohmann and *Gephyrocapsa oceanica* Kamptner, have important implications for the global carbon cycle through processes of photosynthesis, calcification and respiration (Rost and Riebesell, 2004). The 3 noëlaerhabdaceaen genera with extant representatives, *Reticulofenestra, Gephyrocapsa*, and *Emiliania*, are distinguished according to details of coccolith morphology: the elements in the shields of *Reticulofenestra* coccoliths are relatively well calcified such that there are no slits between them, *Gephyrocapsa* coccoliths typically have the same degree of shield calcification as *Reticulofenestra* (i.e., no slits) but also possess a conjunct bridge formed from extended inner tube elements spanning the central area, while *Emiliania* coccoliths do not possess a bridge and have less well calcified shield-elements such that slits exist between them (Young et al., 2003; Bendif and Young, 2014). From fossil evidence, *Gephyrocapsa* is thought to have evolved from *Reticulofenestra* (evolution of the bridge), and *Emiliania*, which first appeared in the fossil record only 291 ka (Raffi et al., 2006) is thought to have evolved from one of the *Gephyrocapsa* species (loss of bridge, reduced calcification of shield elements).

The two most ecologically prominent extant noëlaerhabdaceaens, *E. huxleyi* and *G. oceanica*, have been shown to be genetically distinct, albeit very similar (Hagino et al., 2011; Bendif et al., 2014). In the past 100,000 years, *E. huxleyi* has come to be the numerically dominant member of its functional group in environments as wide ranging as oligotrophic tropical and subtropical open oceans, productive coastal upwelling zones, and the sub-polar ocean, although it only forms striking blooms in certain environments (for example the sub-polar North Atlantic; Paasche, 2001; Raffi et al., 2006). It may now even be invading polar seas (Winter et al., 2014). Several morphotypes of *E. huxleyi* have been defined based on coccolith morphology (coccolith size and degree of calcification; Young and Westbroek, 1991; Young et al., 2003; Hagino et al., 2011). Morphotype A appears broadly distributed, while the less calcified morphotypes B, C, B/C, and O are found principally at higher latitudes, and over-calcified forms (heavily calcified morphotype A, morphotype R) are found principally in upwelling waters of the southern hemisphere. The morphotypes have been shown to be stable over time in culture so it has been assumed that morphotype is at least partly determined genetically (Young and Westbroek, 1991; Paasche, 2001).

All *E. huxleyi* culture strains for which sequence data exists have identical *18S* and *28S rDNA* sequences, but phylogenies based on mitochondrial genes have distinguished two principal *E. huxleyi* clades. The alpha clade consists of isolates originating mainly from tropical, sub-tropical and warm temperate waters, while beta clade isolates originate almost exclusively from colder higher latitude waters (Beaufort et al., 2011; Hagino et al., 2011; Bendif et al., 2014). The different morphotypes are broadly distributed within both mitochondrial clades. The 3′ untranslated region of the GPA gene has been proposed to distinguish partially between morphotypes, as the limited number of B, C, and B/C morphotypes tested so far (7 in total) fell all within one genotype by this marker (Schroeder et al., 2005; Krueger-Hadfield et al., 2014). In contrast, the A morphotype appears to be more genetically diverse in terms of GPA sequences, consistent with its broader distribution. Finally, studies at the level of the whole genome, involving both next-generation resequencing (Illumina) and comparative genome hybridization by microarray, have revealed extensive variability in genome contents and structure between *E. huxleyi* isolates (Kegel et al., 2013; Read et al., 2013; von Dassow et al., 2014), which at least partially correlate with the type of environment from which strains originated.

Of the other extant members of the Noëlaerhabdaceae, *G. oceanica* is typically restricted to mesotrophic sub-tropical and tropical waters (18–30°C), *G. muellerae* Breheret occurs in cooler productive waters (<21°C), while *G. ericsonii* McIntyre and Bé is found in open ocean sub-tropical and tropical waters (12–27°C) (Bollmann, 1997). Less information is available on the distributions of members of the genus *Reticulofenestra*. A “small *Reticulofenestra*” complex including *R. parvula* (Okada and McIntyre) Biekart and *R. punctata* (Okada and McIntyre) Jordan and Young (considered a probable variant of *R. parvula*; Young et al., 2003) is reported to be restricted to central and eutrophic tropical oceans: Pacific (Okada and McIntyre, 1977; Hagino and Okada, 2004), Atlantic (Okada and McIntyre, 1977; Sprengel et al., 2000) and Indian (Takahashi and Okada, 2000), and in Western Mediterranean sea (Cros and Fortuño, 2002; Oviedo et al., 2015), often co-occuring with *G. ericsonii* (Cros and Fortuño, 2002; Hagino and Okada, 2004). *R. sessilis* (Lohmann) Jordan and Young is found in symbiosis with a centric diatom in the equatorial and western Pacific (Okada and McIntyre, 1977), and central and southern Atlantic (Frada et al., 2010), primarily in the deep-photic zone. Lastly, *R. maceria* (Okada and McIntyre) Jordan and Young is known only from its first description, where it was described as “rare” in the Equatorial Pacific (Young et al., 2003). Thus, in contrast to *E. huxleyi*, each of the other noëlaerhabdacean taxa show much more restricted ecological distributions.

Emphasizing their close evolutionary relationship, the two most prominent extant noëlaerhabdaceaens, *E. huxleyi* and *G. oceanica*, have identical *18S rDNA* sequences and differ by only 1 substitution in the *28S rDNA* sequence (Medlin et al., 1996; Edvardsen et al., 2000; Bendif et al., 2014). However, *G. oceanica* consistently separates from *E. huxleyi* based on mitochondrial gene phylogenies (Bendif et al., 2014). Over 500 strains of *E. huxleyi* and 100 strains of *G. oceanica* are maintained in the principal culture collections, but, until recently, no other member of the Noëlaerhabdaceae had (to our knowledge) been successfully isolated into culture by classical methods. This has greatly restricted knowledge of the evolutionary history of *E. huxleyi* and the ability to conduct comparative physiological and genomic studies to understand how this species colonized surface waters of almost the entire ocean. Bendif et al. (2015) reported the results of a morphological and phylogenetic study on the first successful isolation of three clonal cultures of *G. muellerae*. The results of this study challenged the traditional morphology-based taxonomic separation of *Emiliania* from *Gephyrocapsa*, as both nuclear and cytoplasmic gene phylogenies grouped *G. muellerae* within *E. huxleyi*. This study also revealed a major discordance between plastidial and mitochondrial gene phylogenies of strains belonging to *E. huxleyi* and *G. oceanica*. One hypothesis forwarded to explain the discordances between morphological and molecular phylogenetic affiliations and between molecular phylogenies from different genomic compartments was the potential role of hybridization and introgression between closely related species during the expansion of *E. huxleyi*.

**Figure 2:**
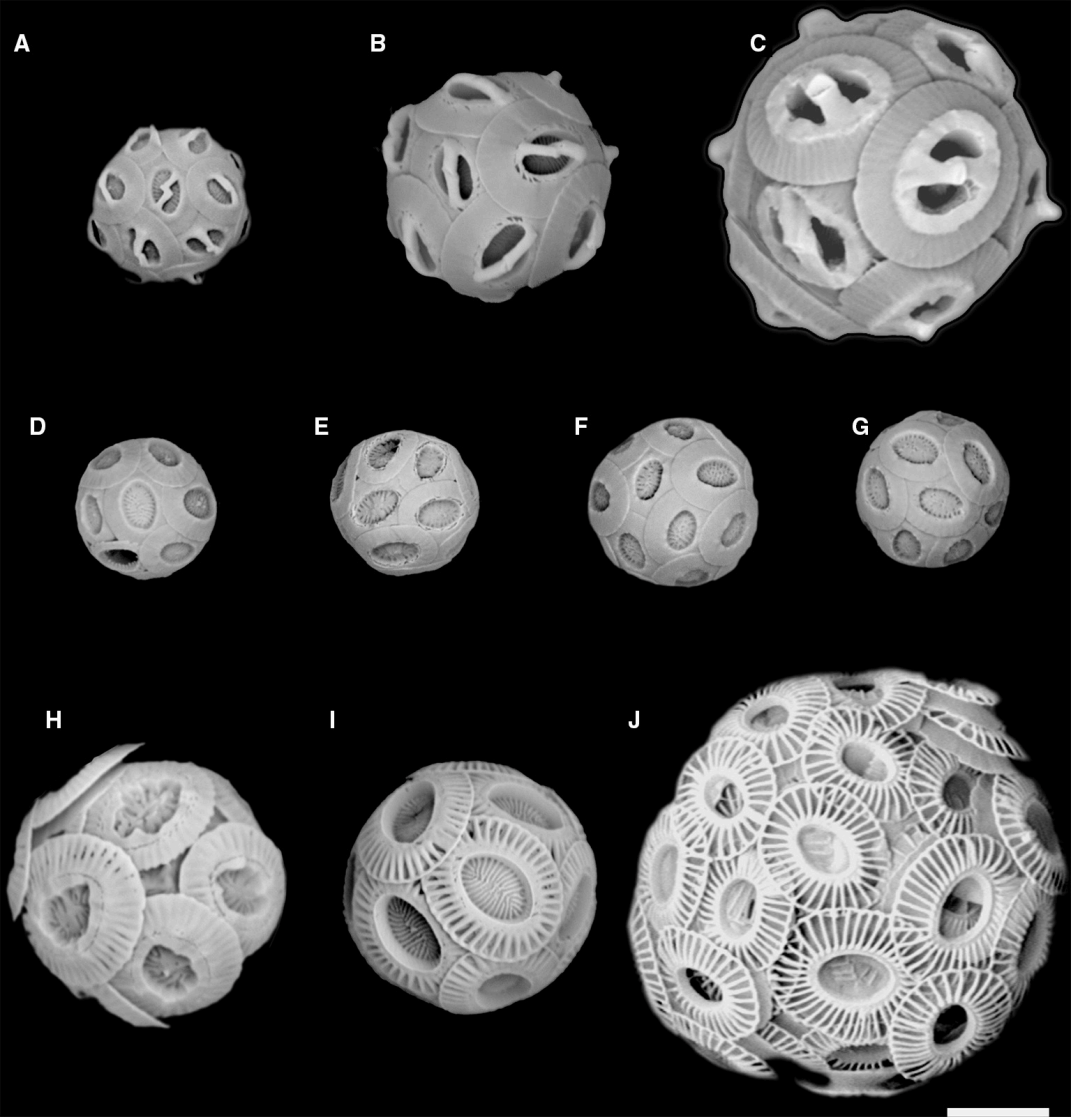
**SEM images of the 5 new Noëlaerhabdaceae isolates and of other representative strains of ***Gephyrocapsa*** and ***Emiliania huxleyi***: (A) ***Gephyrocapsa ericsonii*** RCC4032; (B) ***Gephyrocapsa muellerae***; (C) ***Gephyrocapsa oceanica***; (D) ***Reticulofenestra parvula*** RCC4033; (E) ***R. parvula*** RCC4034; (F) ***R. parvula*** RCC4035; (G) ***R. parvula*** RCC4036; (H) ***E. huxleyi*** morphotype R; (I) ***E. huxleyi*** morphotype A; (J) ***E. huxleyi*** morphotype B (B/C – O)**. Figures 1A,D–G are new isolates. Scale bar = 2 microns.

The isolation into culture of *G. muellerae* was enabled by the use of a novel flow cytometric cell sorting method that uses changes in the polarization of light scatter to detect and sort calcified cells individually (von Dassow et al., 2012). Using the same method here, we successfully obtained multiple new clonal culture strains from samples from the south-east Pacific Ocean, five of which belonged to two noëlaerhabdacean species that, to our knowledge, have never previously been cultured. One is a small (ca. 3 μm coccosphere diameter) *Gephyrocapsa* and the other four are small (ca. 3 μm coccosphere diameter) *Reticulofenestra* (Figures 2A,D–G). Here we present species assignment based on morphological analysis using scanning electron microscopy (SEM), followed by phylogenetic characterization based on nuclear (*18S* and *28S* rRNA), mitochondrial (*cox1* and *cox3*), and chloroplast (*tufA*) genes from these strains.

## Materials and methods

### Origin, culture and morphological characterization of strains

The new noëlaerhabdacean strains were isolated following the procedure described in Bendif et al. (2015). Briefly, surface seawater was collected from the continuous underway water system (CWS) or from Niskin bottles at 5 m or 10 m depth during the NBP1305 cruise aboard the R/V Nathaniel B. Palmer in the south-east Pacific (24th June–22nd July 2013). A volume of 200 ml of seawater was filtered through 20 μm nylon filters (Millipore NY20) to remove larger organisms. To concentrate heavier (mineralized) nanoplankton, 50 ml subsamples of the filtrate were centrifuged for 10 min at 500xg and then 3 min at 1000xg using a swinging bucket rotor. Then, 45 ml of supernatant was discarded, and the remainder combined and centrifuged again to obtain a final volume of 2 ml. Coccolithophores were distinguished in flow cytometry plots by red fluorescence due to chlorophyll and depolarization of forward scatter light (FSC) due to calcite (von Dassow et al., 2012) using an InFlux Mariner cell sorter in an on-board portable laboratory. Individual cells were sorted into individual wells in 96 well PCR plates filled with 100 μl of iK/5 medium (described in von Dassow et al., 2009). In addition to sorting into plates for culturing, 100 FSC-depolarizing cells were also sorted together onto a microscope slide for direct light microscopy observation aboard using a Nikon Eclipse E800 (Nikon, Japan) with Nomaski/DIC optics and a 60x oil objective, and photos were taken with a Spot Insight camera (Diagnostics Instruments). Plates were maintained at 17°C with 50 μmol photons m^−2^ s^−1^ illumination provided by daylight neon tubes with a 14:10h L:D cycle. Successful isolates (including multiple isolates of *E. huxleyi*; Table 1) were transferred into culture flasks after 3–5 weeks and subsequently maintained in the same conditions. Strains were deposited in the Roscoff Culture Collection (RCC, www.roscoff-culture-collection.org).

**Table 1:** **List of strains successfully isolated, and RCC# (if available)**.

**Strain**	**RCC#**	**Sample ID**	**Lat (°)**	**Long (°)**	**Date**	**Depth (m)**	**Temp (°C)**	**Sal**
CHC377	n/a	NBP002	−22.216	−74.227	27-Jun-2013	10	17.9	35.2
CHC384	n/a	NBP002	−22.216	−74.227	27-Jun-2013	10	17.9	35.2
CHC378	n/a	NBP002	−22.216	−74.227	27-Jun-2013	10	17.9	35.2
CHC383	n/a	NBP002	−22.216	−74.227	27-Jun-2013	10	17.9	35.2
CHC450	n/a	NBP203	−16.749	−85.998	09-Jul-2013	60	19.6	35.5
CHC448	n/a	NBP203	−16.749	−85.998	09-Jul-2013	60	19.6	35.5
CHC446	n/a	NBP203	−16.749	−85.998	09-Jul-2013	60	19.6	35.5
CHC445	n/a	NBP203	−16.749	−85.998	09-Jul-2013	60	19.6	35.5
CHC449	n/a	NBP203	−16.749	−85.998	09-Jul-2013	60	19.6	35.5
CHC470	n/a	NBP204	−16.749	−85.998	09-Jul-2013	100	19.1	35.4
CHC462	n/a	NBP367	−21.499	−79.499	13-Jul-2013	75	16.0	34.8
CHC452	n/a	NBP542	−20.769	−70.659	18-Jul-2013	CWS	17.1	35.1
CHC457	n/a	NBP542	−20.769	−70.659	18-Jul-2013	CWS	17.1	35.1
CHC458	n/a	NBP542	−20.769	−70.659	18-Jul-2013	CWS	17.1	35.1
CHC454	n/a	NBP542	−20.769	−70.659	18-Jul-2013	CWS	17.1	35.1
CHC456	n/a	NBP542	−20.769	−70.659	18-Jul-2013	CWS	17.1	35.1
CHC455	n/a	NBP542	−20.769	−70.659	18-Jul-2013	CWS	17.1	35.1
CHC518	n/a	NBP542	−20.769	−70.659	18-Jul-2013	CWS	17.1	35.1
CHC517	n/a	NBP568	−20.748	−70.657	18-Jul-2013	CWS	17.0	35.0
CHC461	n/a	NBP568	−20.748	−70.657	18-Jul-2013	CWS	17.0	35.0
CHC516	RCC4032	NBP568	−20.748	−70.657	18-Jul-2013	CWS	17.0	35.0
CHC527	RCC4033	NBP568	−20.748	−70.657	18-Jul-2013	CWS	17.0	35.0
CHC528	RCC4034	NBP568	−20.748	−70.657	18-Jul-2013	CWS	17.0	35.0
CHC529	RCC4035	NBP568	−20.748	−70.657	18-Jul-2013	CWS	17.0	35.0
CHC530	RCC4036	NBP568	−20.748	−70.657	18-Jul-2013	CWS	17.0	35.0

For scanning electron microscopy (SEM), cells were grown until early exponential phase and then filtered onto polycarbonate filters that were dried in a vacuum desiccator before being sputter coated with a thin layer of Au/Pd. Qualitative observations were made with a Hitachi TM3000 Desktop SEM (Hitachi, Tokyo, Japan) while quantitative observations were made with a Phenom ProX Desktop SEM (Phenom-World, Eindhoven, Netherlands) and measured using ImageJ software (http://imagej.nih.gov/ij/). Morphometric measurements were made with a minimum of 60 isolated coccoliths and coccospheres analyzed per sample. Figure 1 illustrates the key morphological features of noëlaerhabdaceae coccoliths. Following Bollmann (1997), morphometry of Gephyrocapsan coccoliths was based on the coccolith length (the larger of the two ellipsoidal axes) and bridge angle (the angle between the bridge and the long axis; Supplementary Figure 1).

### Characterization of noëlaerhabdacean biogeographies in the southeast pacific

To provide more information on the comparative biogeographic distributions of the Noëlaerhabdaceae, we include an analysis of natural coccolithophore communities from both the NBP1305 cruise and several smaller sampling expeditions in coastal and oceanic sites to the south. Water was collected in Niskin bottles from 5 m and 30 m depth at a total of 6 stations in the strong coastal upwelling center off Punta Lengua de Vaca and Tongoy Bay along the Chilean coast (Lat/Long: −30.25°/−71.69°; −30.18°/−71.59°; −30.12°/−71.62°) from the R/V Stella Maris II on the 13–14 Oct. 2011 and 28 Nov. 2012. At these sites, surface water temperatures ranged from 12.4 to 13.0°C, Water was collected at 5, 40, and 80 m depths from a rented fishing vessel at two sites located 15.5–15.6 km east of Robinson Crusoe Island (Lat/Long: −33.66°/−78.60°; −33.60°/−78.66°) on 1 Nov. 2011, when surface water temperature was 14.8–14.9°C. For Utermöhl counts of total phytoplankton, samples were fixed by adding a 0.1x volume of 10% formaldehyde, 0.5% glutaraldehyde, 100 mM borate, pH 8.7. 100 ml volumes were sedimented and counted with an inverted microscope (CKX41, Olympus). For scanning electron microscopy analysis of coccolithophore community composition, 200 ml samples were filtered directly (without fixation) onto 25 mm 0.4 μm polycarbonate filters and dried. After Au/Pd sputter-coating, a minimum of 80 cells/filter were counted by SEM (Hitachi TM3000 and Quanta 250). Only noëlaerhabdacean coccolithophores, which contributed an average of 94.1 ± 6.9% of all coccolithophores, are reported here. Relative abundances are overlaid on maps of monthly sea surface temperature climatologies (2002–2012) obtained from the Modis Aqua satellite (Feldman and McClain, 2016) and plotted using SeaDAS v7.3 (Baith et al., 2001).

### DNA extraction, amplification, and molecular analysis

Genomic DNA was extracted using the DNeasy Plant mini kit (Qiagen). Partial sequences of the *18S* and *28S* nuclear *rDNA, tufA, cox1* and *cox3* genes were PCR amplified using the primer sets detailed in Bendif et al. (2015). PCRs were performed in a total reaction volume of 25 μL using the GoTaq Polymerase kit (Promega). A standard PCR protocol was used with a T1 thermal cycler (Biometra): 2 min initial denaturation at 95°C, followed by 35 cycles of 30 s at 95°C, 30 s annealing at 55°C and 1 min extension at 72°C. A final 5 min extension step at 72°C was conducted to complete the amplification. Amplification products were controlled by electrophoresis on a 1% agarose gel. The PCR products were sequenced directly on an ABI PRISM 3100 xl DNA auto sequencer (Perkin-Elmer) using the ABI PRISM BigDye Terminator Cycle Sequencing Kit (Perkin-Elmer). Sequences generated were deposited in Genbank (http://www.ncbi.nlm.nih.gov/genbank/) and accession numbers are provided (Supplementary Table 1).

### Phylogenetic analyses

Sequence datasets (including sequences downloaded from Genbank release) were aligned with sequences of other haptophytes (when available) using the online version of the multiple alignment program MAFFT (Katoh and Standley, 2013). Alignments were double checked de visu with SEAVIEW (Gouy et al., 2010). Appropriate models for DNA substitution were estimated with JModeltest2 (Darriba et al., 2012) which selected the same models as those applied in Bendif et al. (2015) for each gene. Nuclear and mitochondrial gene datasets were concatenated separately using SequencMatrix, in order to compare three datasets respective to their genomic compartments: nuclear (*18S* and *28S*), mitochondrial (*cox1* and *cox3*) and plastidial (*tufA*). Phylogenetic trees were constructed using two phylogenetic methods: maximum likelihood (ML) using PhyML implemented in SEAVIEW and Bayesian analysis with Mr. Bayes v3.1.2 (Huelsenbeck and Ronquist, 2001). The robustness of the branching of trees was tested by bootstrapping for the ML inference where bootstrap values were based on 1000 replicates. Bayesian analysis was conducted with two runs of four Markov chains, for at least 5 million generations, sampling every 100th generation to reach minimum likelihood convergence. The burn-in option was set discarding 25% of the 50,000 trees found.

In order to test for phylogenetic discordance between nuclear, mitochondrial and plastidial phylogenies, topology tests were performed using Bootstrap Probabilities (BP), the KH test (Kishino and Hasegawa, 1989), the SH test (Shimodaira and Hasegawa, 1999), and the AU test (Shimodaira, 2002). The null distribution was generated by non-parametric bootstrapping and log likelihood scores of trees constrained by topological conflicts and test values including P values were calculated using CONSEL (Shimodaira and Hasegawa, 2001).

## Results

### Morphospecies identification

Flow cytograms of phytoplankton during cell sorting revealed two distinct groups of cells that depolarized FSC (Supplementary Figure 2A). Together, cells that depolarized FSC represented <1% of nanophytoplankton detected with the cytometer. Direct light microscope examination of sorted cells confirmed that they represented calcified coccolithophores, and appeared to contain two distinct size classes (Supplementary Figure 2B). A total of 25 coccolithophore isolates were successfully established and characterized from the NBP1305 cruise (Table 1). Initial SEM observations confirmed that 20 of these corresponded to *E. huxleyi* while 1 corresponded to the genus *Gephyrocapsa* (Figure 2A) and 4 corresponded to the genus *Reticulofenestra* (Figures 2D–G).

**Figure 3:**
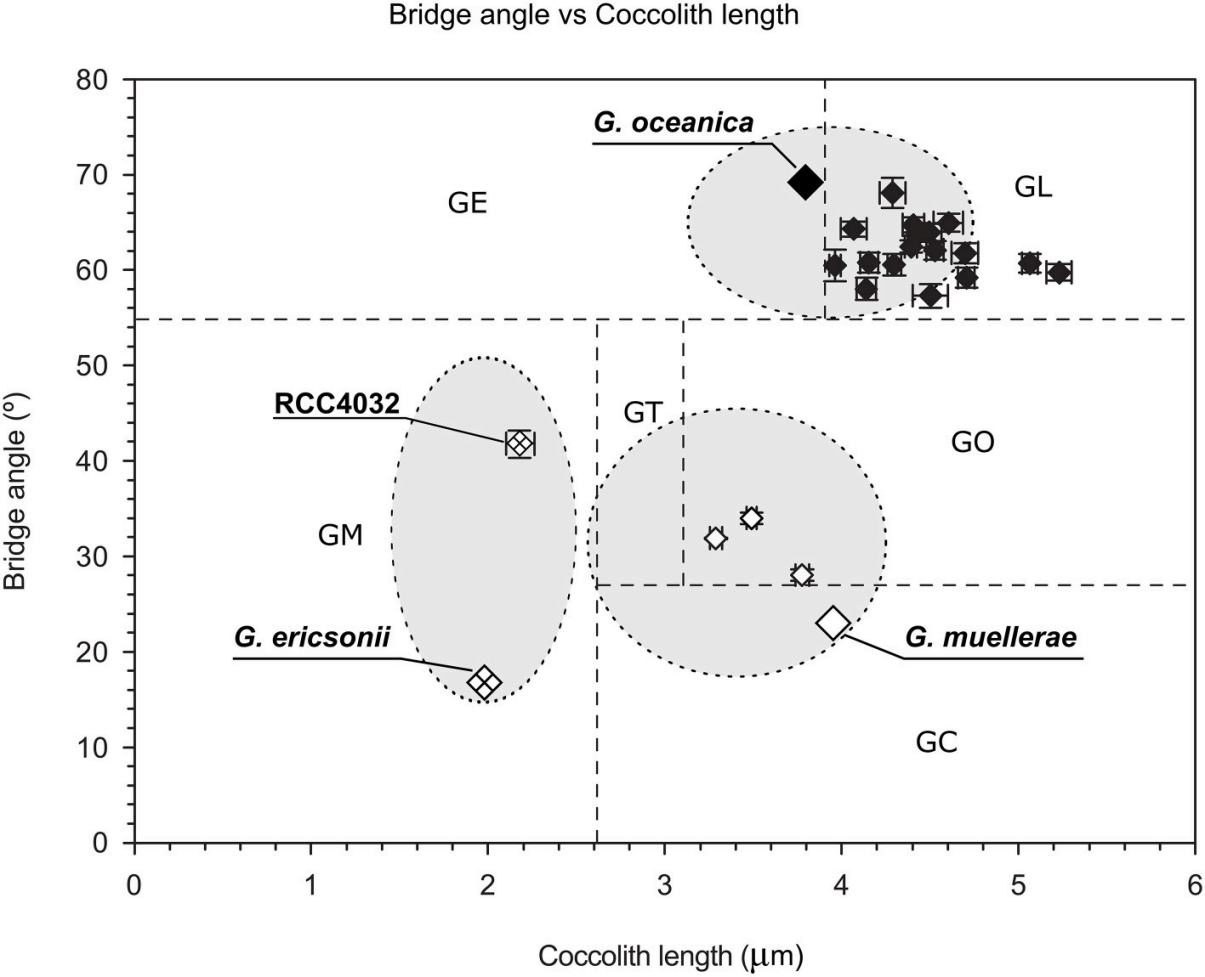
**Scatter plot of mean coccolith length vs. mean bridge angle of ***Gephyrocapsa*** morphotypes defined in Holocene sediment samples (***Gephyrocapsa*** Equatorial (GE), ***Gephyrocapsa*** Oligotrophic (GO), ***Gephyrocapsa*** Transitional (GT), ***Gephyrocapsa*** Cold (GC), ***Gephyrocapsa*** Larger (GL), ***Gephyrocapsa*** Minute (GM)) with values plotted from the original descriptions of (extant) ***Gephyrocapsa ericsonii, Gephyrocapsa muellerae***, and ***Gephyrocapsa oceanica*** (gray area within ellipses represents the range of values generally applied for these species), from ***Gephyrocapsa ericsonii*** isolate RCC4032, and from 3 ***Gephyrocapsa muellerae*** and 16 ***Gephyrocapsa oceanica*** culture strains**. Error bars represent the standard error. After Bollmann (1997) and Young et al. (2003).

McIntyre et al. (1970) introduced a simple concept to distinguish species within *Gephyrocapsa*, with a bridge angle relative to the long axis of the coccolith >45° defining *G. oceanica* and a bridge angle <45° corresponding to *G. caribbeanica* (= *G. muellerae* in modern terminology) when coccolith length is >2.2 microns and to *G. ericsonii* when coccolith length is <2.2 microns. RCC4032 had a mean bridge angle of ca. 41° and mean coccolith length of ca. 2.1 microns (Figure 3; Table 2) and therefore corresponds to *G. ericsonii* according to the criteria of McIntyre et al. (1970). From morphometric measurements of *Gephyrocapsa* coccoliths in globally distributed Holocene sediments, Bollmann (1997) defined 6 different morphological associations within the genus that were tentatively related to existing taxonomic entities and to environmental preferences with respect to temperature and productivity. According to measurements of mean coccolith length and bridge angle, RCC4032 fell into the “*Gephyrocapsa* Minute” (GM) category in this classification (Figure 3). The GM category corresponds to extant *G. ericsonii* (Young et al., 2003). The bridge angle of *G. ericsonii* in the original description (McIntyre and Bé, 1967) is rather low (<20°) relative to that measured for RCC4032 (Figure 3). A number of small (coccolith length <2.4 microns) *Gephyrocapsa* species have been described from Holocene sediments (although in practice these are often grouped into a “small *Gephyrocapsa*” category due to the difficulty of distinguishing fine-scale features in light microscopy) and some of these fossil taxa (e.g., *G. aperta* and notably “*Gephyrocapsa* species form 2” of Samtleben, 1980) have an intermediate bridge angle like RCC4032. However, the only small *Gephyrocapsa* species that is commonly recognized in extant plankton is *G. ericsonii* and we therefore identified RCC4032 as belonging to this species.

**Table 2:** **Average characteristics of isolates measured in this study**.

**Morphospecies**	**RCC#**	**Coccosphere Diameter (**μ**m)**			**Coccolith length (**μ**m)**			**Bridge Angle (**°**)**			**Morphotype**
*Gephyrocapsa ericsonii*	RCC4032	3.65	±0.35	±0.04	2.16	±0.29	±0.04	42.12	±7.36	±0.97	GM
*Reticulofenestra parvula*	RCC4033	3.29	±0.35	±0.04	2.14	±0.25	±0.03		n/a		*R. parvula*
*Reticulofenestra parvula*	RCC4034	3.1	±0.43	±0.06	2.16	±0.29	±0.04		n/a		*R. parvula*
*Reticulofenestra parvula*	RCC4035	3.23	±0.35	±0.04	2.13	±0.25	±0.03		n/a		*R. parvula*
*Reticulofenestra parvula*	RCC4036	3.23	±0.32	±0.04	2.22	±0.23	±0.03		n/a		*R. parvula*

*Standard deviations and standard errors are respectively shown next to the mean of measurements*.

Extant noëlaerhabdaceans with no bridge and no slits in the shield are classified in the genus *Reticulofenestra*. The four new culture strains RCC4033, RCC4034, RCC4035, and RCC4036 had near identical coccolith morphologies that corresponded (no bridge, no slits) to that of the extant species *R. parvula* (Okada and McIntyre, 1977; Biekart, 1989; Young et al., 2003). Average coccolith length for the four strains varied from 2.14 to 2.2 microns. These values slightly exceed the coccolith length reported in the original description of *R. parvula* (<2 microns; Okada and McIntyre, 1977), but this difference is insignificant when standard deviation values (phenotypic variability within strains) are taken into account. Coccospheres of these strains measured between 3.10 and 3.29 microns in our culture conditions (Table 2).

### Biogeography of noëlaerhabdaceae in the south-east pacific

**Figure 4:**
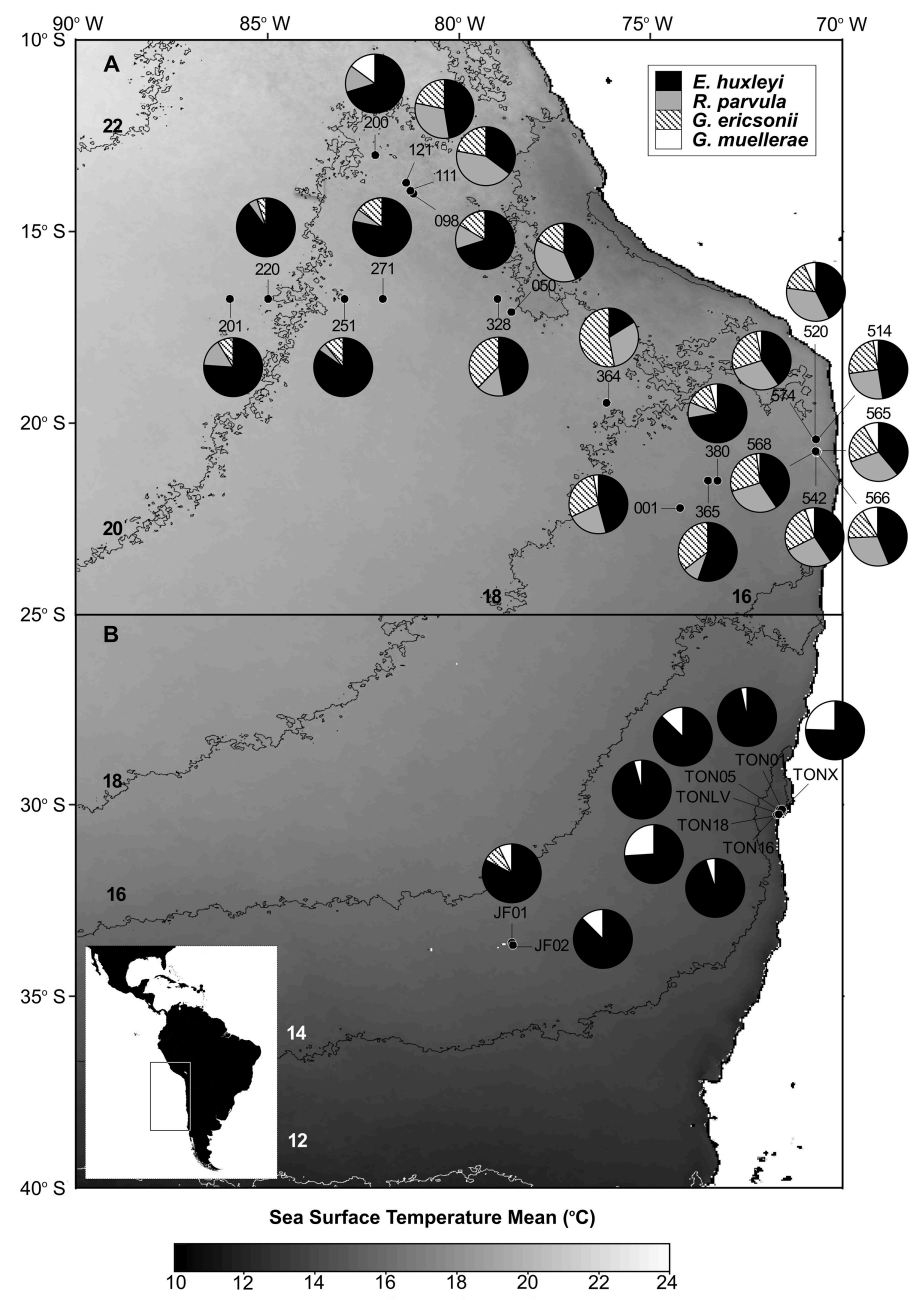
**Pie charts showing the distribution and relative abundances of ***Reticulofenestra parvula***, ***Gephyrocapsa ericsonii***, ***Gephyrocapsa muellerae***, and ***Emiliania huxleyi*** in the eastern South Pacific**. **(A,B)** maps show the satellite sea surface temperature (SST) monthly climatology from Modis Aqua for July (2002–2014) and October (2002–2014), respectively. The sampling stations are depicted in black dots. Each 2°C SST isopleth is shown and labeled in black or white.

The relative abundances of *E. huxleyi, G. ericsonii, G. muellerae*, and *R. parvula* in south-east Pacific waters from samples taken in 2011–2012 are presented in Figure 3. *E. huxleyi* was ubiquitous in all samples (from ca. 11°S to ca. 34°S) and often dominated assemblages. *G. ericsonii*, which is considered a warm water species with maximum abundances between 13 and 22°C (Okada and McIntyre, 1979), was present in almost all samples >16°C (and absent below 16°C), closely matching distributions expected from earlier studies McIntyre et al., 1970; Okada and McIntyre (1977). The water temperature of the sample from which *G. ericsonii* RCC4032 was isolated was 17°C (Table 1). *R. parvula* almost always co-occurred with *G. ericsonii* in relatively warm water (Figure 4; Table 1). In contrast, *G. muellerae* has been associated with cooler waters (<21°C) and in our survey was limited to waters <17° C (Figure 4).

### Phylogenetic position of the new strains and cyto-nuclear discordance of *Emiliania* and *Gephyrocapsa* strains

**Figure 5:**
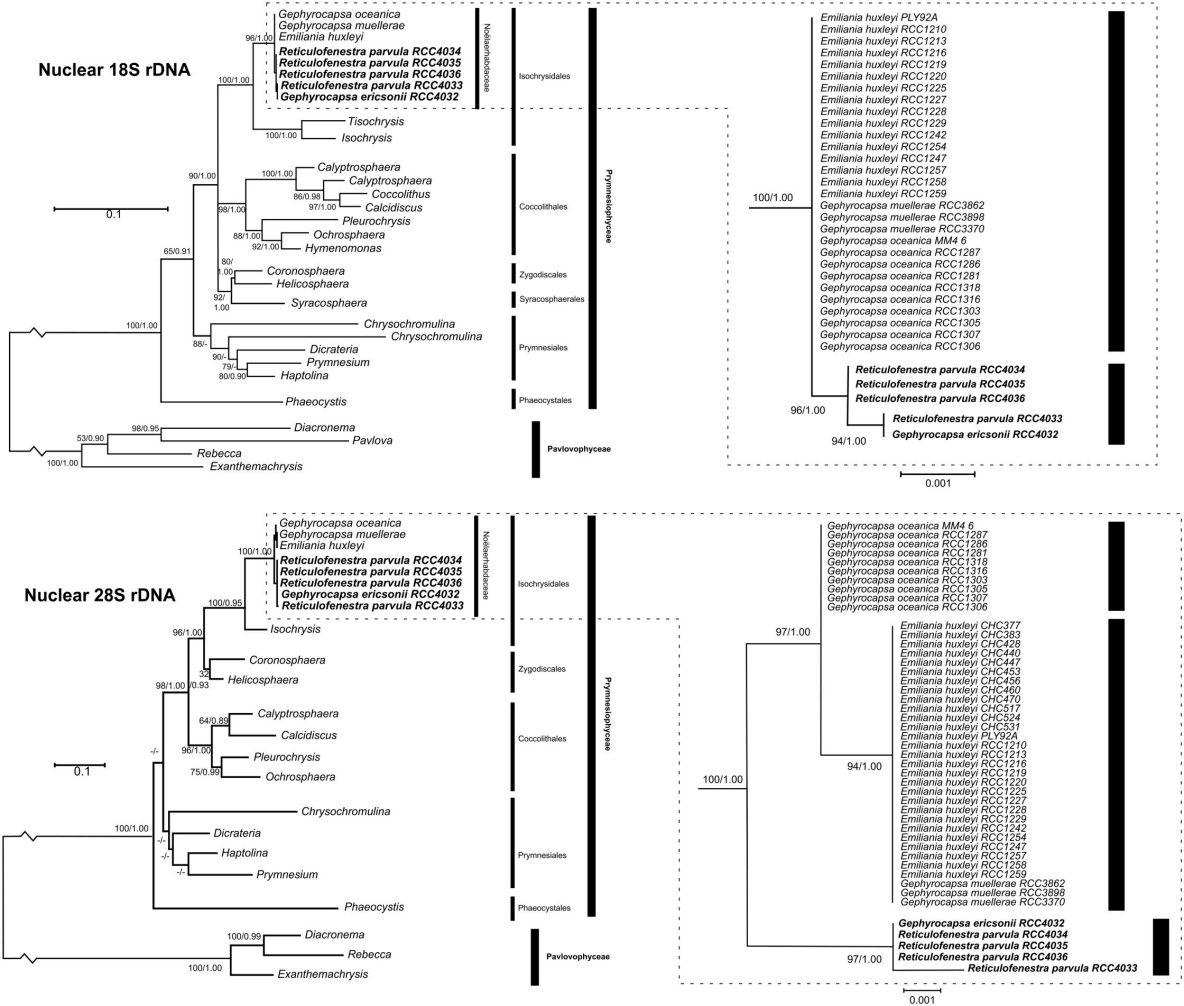
**Molecular phylogeny of haptophytes inferred from comparisons of partial nuclear ***18S*** (top left) and ***28S rDNA*** (bottom left) sequences with detail on subtrees of the Noëlaerhabdaceae (top right, bottom right)**. Support values at each node are presented for ML/Bayes analyses. Bootstrap values larger than 50 and posterior probabilities larger than 0.80 are shown. Lesser values are represented by “–.”

The 5 new strains were examined by sequencing partial fragments of nuclear *18S* and *28S rDNA*, plastidial *tufA*, and mitochondrial *cox1* genes, and the complete mitochondrial *cox3* gene (Table 3). Comparison with haptophyte nuclear *18S* and *28S rDNA* sequences retrieved from Genbank confirmed the phylogenetic position of the *G. ericsonii* and *Reticulofenestra* strains within the Noëlaerhabdaceae, together forming a distinct clade within the *Gephyrocapsa* complex (Figure 5). Ribosomal sequences differed by 3 nucleotides (1bp for *18S* and 2bp for *28S*) and 4 nucleotides (1bp for *18S* and 3bp for *28S*) between this clade and respectively *G. oceanica* and *E. huxleyi*/*G. muellerae* (Supplementary Table 2).

**Table 3:** *****P***-values and likelihood scores obtained from the topology tests (BP, Bootstrap Probabilities, KH, Kishino-Hasegawa, SH, Shimodaira-Hasegawa, WSH, Weighted Shimodaira-Hasegawa; and AU, Approximately Unbiased)**.

	**18S+28S vs. 18S+28S**	**18S+28S vs. cox1+cox3**	**18S+28S vs. tufA**	**cox1+cox3 vs. 18S+28S**	**cox1+cox3 vs. cox1+cox3**	**cox1+cox3 vs. tufA**	**tufA vs. 18S+28S**	**tufA vs. cox1+cox3**	**tufA vs. tufA**
BP	0.989	**0**	**0**	**0**	1	**0**	**0**	**0**	1
KH	1	**0.021**	**0.04**	**0**	1	**0**	**0**	**0**	1
SH	1	**0.021**	**0.04**	**0**	1	**0**	**0**	**0**	1
WSH	1	**0.032**	**0.04**	**0**	1	**0**	**0**	**0**	1
AU	0.994	**0**	**0**	**0**	0.235	**0**	**0**	**0**	0.235
ln	−3114.161	−3217.96	−3192.395	−3213.426	−2301.843	−2819.113	−1822.457	−1667.534	−1259.645

*Values in bold indicate rejection of the null hypothesis with 95% confidence*.

The topologies of *cox1* and *cox3* phylogenies were very similar to each other, with *G. ericsonii* and *R. parvula* sequences clustered within the alpha haplo-group of *E. huxleyi* for both genes (Supplementary Figures 3, 4). For both *cox1* and *cox3*, sequences of *G. ericsonii* (strain RCC4032) were identical to *R. parvula* strain RCC4033 and some *E. huxleyi* strains, but differed from the three other *R. parvula* strains. Mitochondrial sequences of the latter 3 strains clustered together within a sub-group formed with other *E. huxleyi* strains that included RCC1242 [= CCMP1516, the first strain from which a genome assembly has been published Read et al., 2013], although these 3 *R. parvula* strains did still separate from the *E. huxleyi* strains with 85% ML bootstrap and 1.00 Bayesian posterior probability support. Plastidial *tufA* sequence phylogenies (Supplementary Figure 5) clustered *G. ericsonii* and *R. parvula* within the *tufAI* haplo-group, previously defined by Bendif et al. (2014) and composed of *E. huxleyi* and *G. oceanica strains*. The four *R. parvula* were identical and formed a sub-group with *E. huxleyi* strain RCC1242 (CCMP1516) rather than with *G. ericsonii*, which was identical to other *E. huxleyi* and *G. oceanica* strains within the *tufAI* haplo-group, differing by 2 substitutions from the *R. parvula* group.

The phylogenetic reconstructions were rooted with outgroups for which sequences were retrieved from Genbank and from transcriptomic data from the Marine Microbial Eukaryote Transcriptome Sequencing Project (MMETSP; http://marinemicroeukaryotes.org/project_organisms; Keeling et al., 2014). Rooting was more effective for ribosomal and plastidial phylogenies than for mitochondrial phylogenies due to the high degree of divergence between noëlaerhabdaceaen mitochondrial sequences and those from other (relatively distantly related) haptophytes available in public databases. This lack of resolution in rooting the mitochondrial trees was more pronounced for *cox3* than for *cox1*, which resulted in a lack of phylogenetic signal coming from excess in rate of substitution. The ribosomal, plastidial and *cox1* phylogenies all gave high support for rooting the Noëlaerhabdaceae with a last common ancestor (LCA), whereas the *cox3* reconstruction rooted the Noëlaerhabdaceae at the base of the alpha clade. The tree reconstructed from the concatenation of *cox1* and *cox3* sequences appeared to have been more influenced by the *cox3* than by the *cox1* phylogenetic signal. This matter could be resolved by expanding the comparison to more mitochondrial genes and haptophyte taxa, for which data are still lacking.

**Figure 6:**
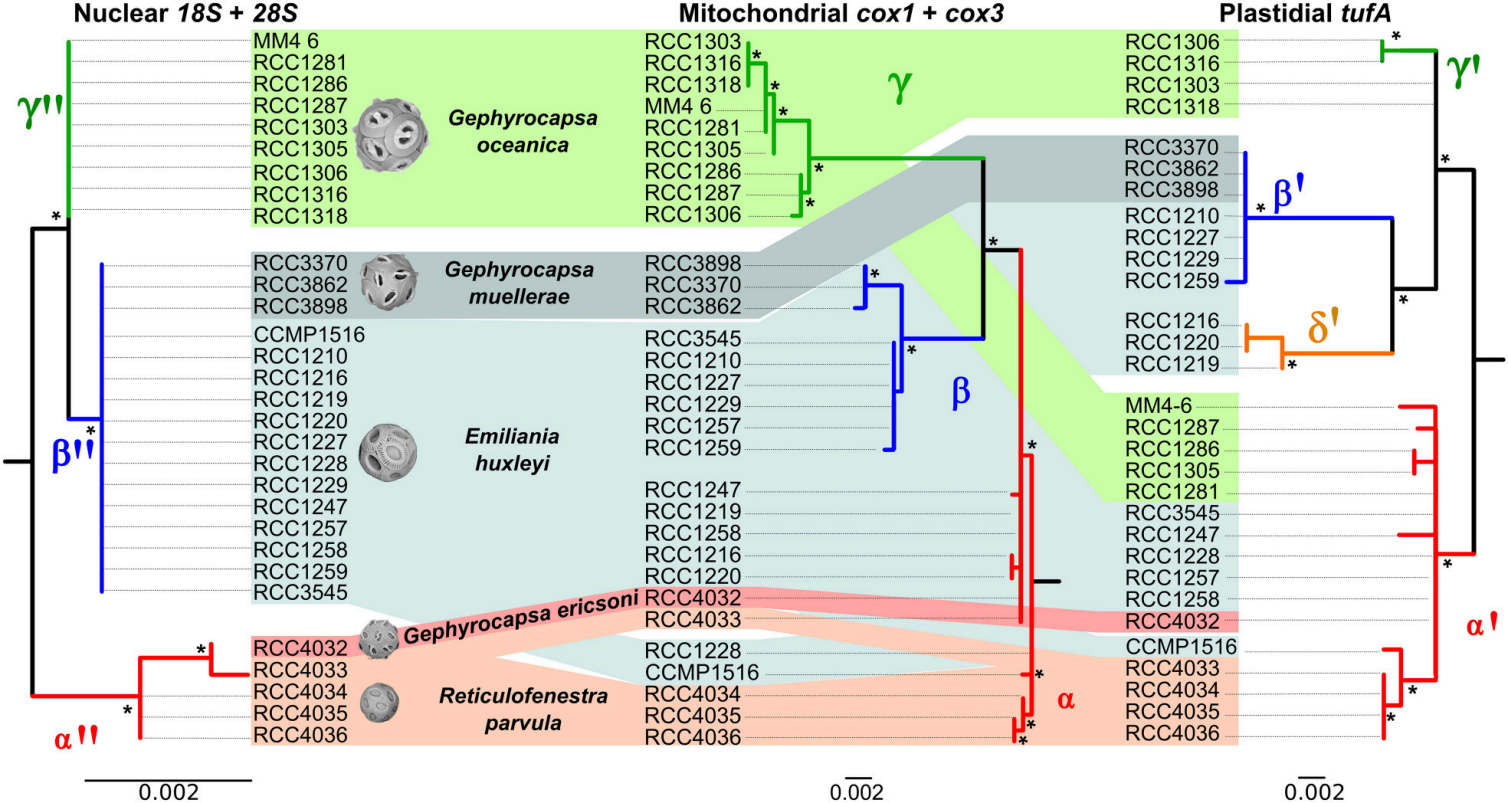
**Comparison of nuclear, mitochondrial and plastidial phylogenies**. Incongruent positions are indicated with dashed lines. Support value for each node is presented for ML. Bootstrap values larger than 50 are shown with an asterisk.

Comparison of ribosomal, mitochondrial and plastidial marker phylogenies revealed topological incongruency in grouping some *E. huxleyi* and *G. oceanica* strains, depicting different phylogenetic signals (Figure 6). Topology tests significantly rejected any congruence (*P*-values below 0.05) between the 3 phylogenies (Table 3). By taking the ribosomal phylogeny as reference, the position of the *G. ericsonii* and *R. parvula* strains remained similar (i.e., basal with respect to the root) amongst the three genomic compartments and could therefore reflect the same cladogenetic episode. When compared to mitochondrial markers, *E. huxleyi* alpha clade (warm group) strains showed discordant positions, clustering with the new *G. ericsonii* and *R. parvula* isolates. When compared to the plastidial phylogeny, some *E. huxleyi* and *G. oceanica* strains clustered with the new *G. ericsonii* and *R. parvula* isolates, showing discordance with the nuclear *rDNA* phylogeny (in the case of both *E. huxleyi* and *G. oceanica*) and with the mitochondrial phylogeny (in the case of the *G. oceanica* strains). Also, 3 *E. huxelyi* strains (all isolated from the Tasman Sea) clustered together in the separate *tufAII* clade, showing discordance with their position in the mitochondrial phylogeny. By considering only concordant positions between the 3 trees, we were able to define three clades for the ribosomal phylogeny: (1) “alpha,” where all the new *G. ericsonii* and *R. parvula* strains clustered together, (2) “beta,” where *E. huxleyi* and *G. muellerae* clustered together, and (3) “gamma,” a clade exclusively composed of *G. oceanica* (Figure 6). Therefore, a new nomenclature was proposed for the plastidial clades to highlight concordances and discordances with the nuclear and mitochondrial phylogenies (Figure 6), with (1) alpha' for *tufAI*, (2) beta' for *tufAIII*, (3) gamma' for *tufAGO*, and (4) delta' for *tufAII*.

## Discussion

Morphometric analysis of the coccoliths and coccospheres of the new noëlhaerbdacean strains reported here indicates that they correspond to *Gephyrocapsa ericsonii* (RCC4032) and *Reticulofenestra parvula* (RCC4033, RCC4034, RCC4035, and RCC4036), which to our knowledge have never previously been maintained in culture. This study represents the first combined morphological and genetic analysis on these species. The results provide insights into the evolutionary history of the commonly studied coccolithophores *E. huxleyi* and *G. oceanica*.

### Evolutionary history of extant noëlaerhabdaceae

Unlike the case for *G. muellerae* reported by Bendif et al. (2015), the nuclear *18S* and *28S rDNA* sequences of the new *G. ericsonii* isolate were distinct from those of *E. huxleyi* and *G. oceanica* (Figure 5). *E. huxleyi* has been suggested to have been likely to have originally evolved from *G. ericsonii* (McIntyre, 1970) due to similarity in coccolith size and due to the fact that a form of *G. ericsonii* (often referred to as *G. protohuxleyi*) exists that has slits between shield elements, like *E. huxleyi*. Our results indicate that *E. huxleyi* has a closer genetic relationship with *G. oceanica* and particularly with *G. muellerae* than with *G. ericsonii* (Figure 6).

Our results also suggest that the evolutionary history of the group was more complex than previously thought. If the LCA of extant noëlaerhadceaens was *Reticulofenestra*-like (as is commonly assumed), our phylogenetic reconstruction indicates that bridge formation occurred independently at least twice (in *G. ericsonii* and in the LCA of *G. oceanica* and *G. muellerae*). If, on the other hand, the LCA was *Gephyrocapsa*-like, bridge loss occurred independently at least twice (in *R. parvula* and *E. huxleyi*). This means that either *Gephyrocapsa* or *Reticulofenestra* is paraphyletic or polyphyletic, highlighting the fact that morphological changes such as bridge gain and loss are probably much more evolutionarily dynamic within the Noëlaerhabdaceae than previously thought. From the data produced in this study, the only taxonomic revision that we can confidently apply in order to address this problem is the transfer of *R. parvula* to *Gephyrocapsa* (see Taxonomic Appendix). A strong case could be made, however, for transferring all *Reticulofenestra* species (extant and fossil) to *Gephyrocapsa* (which has nomenclatural priority). The transfer of *Emiliania* to *Gephyrocapsa* has already been formally proposed (Reinhardt, 1972) and supported (Bendif et al., 2015). While the lumping of species from three genera into one genus could be considered as an undesirable loss of taxonomic resolution, the alternative is to propagate a system that clearly misrepresents the evolutionary relationships between some (possibly most) of the organisms in the lineage. One or more independent lineages of organisms with coccolith morphology corresponding to *Reticulofenestra* (i.e., phylogenetically distinct from the extant *R. parvula* represented by 4 strains in the present study) might have existed in the past, and the other extant *Reticulofenestra* species, *R. sessilis*, could prove to be a living representative of one of these, but even if this were determined, the current distinction of genera based on morphological criteria of coccoliths would be unworkable.

The mitochondrial (*cox1* and *cox3*) and plastidial (*tufA*) sequences obtained from our new culture isolates of *G. ericsonii* and *R. parvula* indicate a complex pattern of cyto-nuclear genetic incongruence. In contrast to the clear distinction in nuclear *rDNA* sequences, the mitochondrial and plastidial sequences of these two species intermingled with those of *E. huxleyi* (Figure 6). Mitochondrial markers from both species grouped within the alpha *E. huxleyi* clade. This is in contrast to mitochondrial markers from *G. muellerae* that grouped with the beta *E. huxleyi* clade, and with *G. oceanica* that forms a distinct clade in mitochondrial phylogenies (Bendif et al., 2015). Plastidial markers from *G. ericsonii* and *R. parvula* grouped in the alpha' clade with some (but not all) *E. huxleyi* and *G. oceanica* strains (Figure 6). These results provide further evidence of a complex pattern of reticulate evolution within this lineage that corresponds to ecological associations: *G. muellerae* and members of the beta *E. huxleyi* clade, which group together in mitochondrial phylogenies, are both predominantly distributed in cool temperate to sub-polar waters. Likewise, *G. ericsonii* and *R. parvula* are distributed in the same (warmer water) zones as strains from the alpha *E. huxleyi* clade with which they cluster in mitochondrial phylogenies.

The generally more conservative nuclear *rDNA* genes revealed a clear genetic distinction between *G. ericsonii*/*R. parvula* and the *E. huxleyi* strains in the alpha mitochondrial clade, a distinction that is not present between *G. muellerae* and strains of the beta *E. huxleyi* clade. This is not consistent with the possibility of multiple independent origins of *E. huxleyi* from different *Gephyrocapsa* lineages and therefore strengthens support for the hypothesis of introgressive hybridization being responsible for the phylogenetic patterns observed. Our results suggest that introgressive hybridization may have occurred (and still be occurring) between *G. ericsonii* and *R. parvula*, although the very high level of genetic similarity between these species may simply reflect a very recent common origin. Both of these potential situations could be evoked to explain the interesting fact that there is a closer relationship between RCC4032 (*G. ericsonii*) and RCC4033 (*R. parvula*) than between these two strains and the other *R. parvula* isolates (RCC4034, RCC4035, and RCC4036) in nuclear, mitochondrial and plastidial phylogenies. The results also suggest that introgressive hybridization might have occurred (and might still be occurring) between *G. ericsonii*/*R. parvula* and the *E. huxleyi* and *G. oceanica* populations with which they share an ecological range.

Hybridization appears to play complex roles in range expansions and invasions. Hybrids might show increased invasiveness potential over parent species due to increased genetic variability, hybrid vigor (heterosis), and reduced genetic load (Ellstrand and Schierenbeck, 2000; Hovick and Whitney, 2014). Meanwhile, populations of a species invading a new habitat may obtain alleles favored in the new conditions through introgressive hybridization with closely related native species (Rieseberg et al., 2007). Hybrids often exhibit sterility or reduced fertility due to chromosome incompatibilities or rearrangements. This can contribute to decline or even extinction of native species when swamped by hybridization with large populations of the invader. In addition, increased reliance on parthenogenesis can stabilize heterosis in the invading hybrid. Intriguingly, loss of key genes involved in the life cycle and genomic structural rearrangements have been documented in some alpha clade *E. huxleyi* (von Dassow et al., 2014), partially explaining the high genome variability documented in this species by both genome re-sequencing (Read et al., 2013) and comparative genome hybridization (Kegel et al., 2013). In this context, *E. huxleyi* currently has the broadest ecological distribution despite having appeared more recently in the fossil record compared to the other *Gephyrocapsa* forms. Thus, we presume that *E. huxleyi* has played the role of invader, acquiring plastids and mitochondria from older endemics as it expanded.

## Concluding remarks

A major limitation in the study of marine microbes is that a large fraction of their diversity has not been successfully cultured (Rappe and Giovannoni, 2003; Massana, 2015). This study, combined with the previous first successful isolation of *G. muellerae* (Bendif et al., 2015), shows that single-cell sorting using novel detection mechanisms to target specific functional groups can be highly successful at enhancing the cultured diversity of certain groups. In these two studies, coccolithophores represented less than 1% of total nanophytoplankton cells in environmental samples and yet were successfully isolated into clonal culture, including three previously uncultured species in addition to *E. huxleyi* which is readily cultured by classical techniques. Analysis of these newly cultured species offers novel and unexpected insights into the evolutionary mechanisms acting in eukaryotic phytoplankton meta-populations.

Resolving how hybridization may have facilitated the colonization of the global ocean by *E. huxleyi* will require a combination of comparative physiological and genomic studies. An interesting note is that the published coccolithophore genome assembly (Read et al., 2013) comes from *E. huxleyi* strain CCMP1516 (= RCC1242). In both mitochondrial and plastidial phylogenies, this strain is even more closely affiliated with the *R. parvula* strains reported here than is *G. ericsonii*, and so its genome assembly might already record recent hybridization history.

Concurrently, extensive genome-scale sequencing would inform on how each morphospecies contributes to the *Emiliania/Gephyrocapsa* pan-genome, while identifying which nuclear, mitochondrial or chloroplast genes confer this complex the adaptive potential to radiate into very distinct ocean habitats.

At a taxonomic level, the combined results presented here of sequencing of markers from different genomic compartments provide further support for considering the extant Noëlaerhabdaceae as a complex of interacting species in which existing taxonomic boundaries reflect neither evolutionary nor ecological relationships. This represents an interesting case study on the difficulty of defining a unified taxonomic concept for protists, even when comparing relatively closely related organisms.

## Author contributions

PV and GV designed flow-cytometer procedures, PV performed cell isolation, EB, PV, IP identified and maintained new strains in culture, FDR performed biogeographic study, EB and JY conducted coccolith measurements, EB and DT performed genetic analyses, EB, PV and IP analyzed data; all authors participated in writing and editing the manuscript.

## Funding

This work was supported by the Comisión Nacional de Investigación Científica y Tecnológica of the Chilean Ministry of Education (FONDECYT Regular grants 1110575 and 1141106 and grant CONICYT USA 20120014 to PD and a doctoral fellowship CONICYT-PCHA/Doctorado Nacional/2013-21130158 to FDR), the European Research Council under the European Community's Seventh Framework Programme (EC-FP7) via a Marie Curie Intra-European Fellowship (grant FP7-PEOPLE-2012-IEF; EB), the ASSEMBLE program (grant 227799; EB, IP) and via the French ANR project EMBRC-France (IP), and the International Research Network “Diversity, Evolution and Biotechnology of Marine Algae” (GDRI N° 0803; IP, PD).

### Conflict of interest statement

The authors declare that the research was conducted in the absence of any commercial or financial relationships that could be construed as a potential conflict of interest.

## Taxonomic appendix

*Gephyrocapsa parvula* (Okada and McIntyre) Bendif, Probert, Young and von Dassow comb. nov.

BASIONYM: *Crenalithus parvulus* Okada and McIntyre, *Micro. Pal*. 23: 6-7, pl. 2, figs. 1-2, 1977.

SYNONYM: *Reticulofenestra parvula* (Okada and McIntyre) Biekart, *Proc. Kon. Nederl. Akad. Wet*. 92: pl. 40, 1989.
