# Supplementary material for: Recent Reticulate Evolution in the Ecologically Dominant Lineage of Coccolithophores
Source: Front Microbiol. 2016 May 24;7:784. doi: 10.3389/fmicb.2016.00784 (PMC4877371; doi:10.3389/fmicb.2016.00784)
Supplement: Supplementary file 1 [file Table1.DOC]

Supplementary Material

**Further evidence for recent reticulate evolution in the ecologically dominant lineage of coccolithophores**

El Mahdi Bendif^1*^, Ian Probert^2,3^, Francisco Díaz-Rosas ^4,5,6^, Daniela Thomas^4,5,6^, Ger van den Engh^7^, Jeremy R. Young^8^ and Peter von Dassow^4.5.6 *^

^1^ Marine Biological Association of the UK, Plymouth, UK

^2^ Université Pierre et Marie Curie (Paris VI), Roscoff Culture Collection, Station Biologique de Roscoff, Roscoff, France

^3^ Centre National de la Recherche Scientifique, FR2424, Station Biologique de Roscoff, Roscoff, France

^4^ Facultad de Ciencias Biológicas, Pontificia Universidad Católica de Chile, Santiago, Chile

^5^ Instituto Milenio de Oceanografía, Chile

^6^ UMI 3614, Evolutionary Biology and Ecology of Algae, CNRS-UPMC Sorbonne Universités, PUCCh, UACH, Station Biologique de Roscoff, Roscoff, France

^7^ Center for Marine Cytometry, Concrete, WA, USA

^8^ Departments of Earth Sciences, University College London, London, UK

*** Correspondences:**

**Peter von Dassow**

**pvondassow@bio.puc.cl**

**El Mahdi Bendif**

**elmhidi@gmail.com**

**Ian Probert**

**probert@sb-roscoff.fr**

**1 Supplementary Table**

Supplementary Table 1. Genbank accession number of new isolates

| Morphospecies | Strain | RCC# | tufA | 18S | 28S | cox1 | cox3 |
| --- | --- | --- | --- | --- | --- | --- | --- |
| *Emiliania huxleyi* | CHC377 | n/a | KX056531 |  | KX056537 | KX056555 | KX056580 |
| *Emiliania huxleyi* | CHC378 | n/a |  |  |  | KX056556 | KX056581 |
| *Emiliania huxleyi* | CHC383 | n/a |  |  | KX056538 | KX056557 | KX056582 |
| *Emiliania huxleyi* | CHC384 | n/a |  |  |  | KX056558 | KX056583 |
| *Emiliania huxleyi* | CHC428 | n/a |  |  | KX056539 |  | KX056584 |
| *Emiliania huxleyi* | CHC440 | n/a |  |  | KX056540 |  |  |
| *Emiliania huxleyi* | CHC445 | n/a |  |  |  | KX056559 | KX056585 |
| *Emiliania huxleyi* | CHC446 | n/a |  |  |  | KX056560 | KX056586 |
| *Emiliania huxleyi* | CHC447 | n/a |  |  | KX056541 |  |  |
| *Emiliania huxleyi* | CHC448 | n/a |  |  |  | KX056561 | KX056587 |
| *Emiliania huxleyi* | CHC449 | n/a |  |  |  | KX056562 | KX056588 |
| *Emiliania huxleyi* | CHC450 | n/a |  |  |  | KX056563 | KX056589 |
| *Emiliania huxleyi* | CHC452 | n/a |  |  |  | KX056564 | KX056590 |
| *Emiliania huxleyi* | CHC453 | n/a |  |  | KX056542 |  | KX056591 |
| *Emiliania huxleyi* | CHC454 | n/a |  |  |  | KX056565 | KX056592 |
| *Emiliania huxleyi* | CHC455 | n/a |  |  |  | KX056566 | KX056593 |
| *Emiliania huxleyi* | CHC456 | n/a |  |  | KX056549 | KX056567 | KX056594 |
| *Emiliania huxleyi* | CHC457 | n/a |  |  |  | KX056568 | KX056595 |
| *Emiliania huxleyi* | CHC458 | n/a |  |  |  | KX056569 | KX056596 |
| *Emiliania huxleyi* | CHC460 | n/a |  |  | KX056543 |  |  |
| *Emiliania huxleyi* | CHC461 | n/a |  |  |  | KX056570 | KX056597 |
| *Emiliania huxleyi* | CHC462 | n/a |  |  |  | KX056571 | KX056598 |
| *Emiliania huxleyi* | CHC470 | n/a | KX056529 |  | KX056544 | KX056572 | KX056599 |
| *Gephyrocapsa ericsonii* | CHC516 | RCC4032 | KX056530 | KX056532 | KX056550 | KX056575 | KX056602 |
| *Emiliania huxleyi* | CHC517 | n/a |  |  | KX056545 | KX056573 | KX056600 |
| *Emiliania huxleyi* | CHC518 | n/a |  |  | KX056546 | KX056574 | KX056601 |
| *Emiliania huxleyi* | CHC524 | n/a |  |  | KX056547 |  |  |
| *Reticulofenestra parvula* | CHC527 | RCC4033 | KX056528 | KX056533 | KX056551 | KX056576 | KX056603 |
| *Reticulofenestra parvula* | CHC528 | RCC4034 | KX056525 | KX056534 | KX056552 | KX056577 | KX056606 |
| *Reticulofenestra parvula* | CHC529 | RCC4035 | KX056526 | KX056535 | KX056553 | KX056578 | KX056604 |
| *Reticulofenestra parvula* | CHC530 | RCC4036 | KX056527 | KX056536 | KX056554 | KX056579 | KX056605 |
| *Emiliania huxleyi* | CHC531 | n/a |  |  | KX056548 |  |  |

Supplementary Table 2. Substitution matrix for ribosomal gene markers (*18S* + *28S* *rDNA*).

|  | *G. ericsonii RCC4032* | *R. parvula RCC4033* | *R. parvula RCC4034* | *R. parvula RCC4035* | *R. parvula RCC4036* | *E. huxleyi/G. muellerae* | *G. oceanica* |
| --- | --- | --- | --- | --- | --- | --- | --- |
| *G. ericsonii RCC4032* | 0 (= 0 + 0) |  |  |  |  |  |  |
| *R. parvula RCC4033* | 1 (= 0 + 1) | 0 (= 0 + 0) |  |  |  |  |  |
| *R. parvula RCC4034* | 1 (= 1 + 0) | 2 (= 1 + 1) | 0 (= 0 + 0) |  |  |  |  |
| *R. parvula RCC4035* | 1 (= 1 + 0) | 2 (= 1 + 1) | 0 (= 0 + 0) | 0 (= 0 + 0) |  |  |  |
| *R. parvula RCC4036* | 1 (= 1 + 0) | 2 (= 1 + 1) | 0 (= 0 + 0) | 0 (= 0 + 0) | 0 (= 0 + 0) |  |  |
| *E. huxleyi/G. muellerae* | 5 (= 2 + 3) | 6 (= 2 + 4) | 4 (= 1 + 3) | 4 (= 1 + 3) | 4 (= 1 + 3) | 0 (= 0 + 0) |  |
| *G. oceanica* | 4 (= 2 + 2) | 5 (= 2 + 3) | 3 (= 1 + 2) | 3 (= 1 + 2) | 3 (= 1 + 2) | 1 (= 0 + 1) | 0 (= 0 + 0) |

**
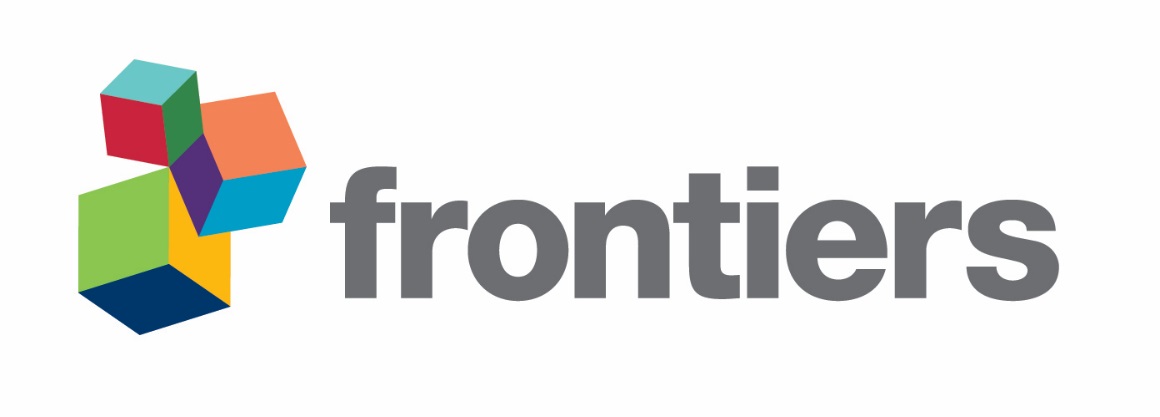
**
