# Supplementary material for: Recent Reticulate Evolution in the Ecologically Dominant Lineage of Coccolithophores
Source: Front Microbiol. 2016 May 24;7:784. doi: 10.3389/fmicb.2016.00784 (PMC4877371; doi:10.3389/fmicb.2016.00784)
Supplement: Supplementary file 2 [file DataSheet1.doc]

Supplementary Material

**Further evidence for recent reticulate evolution in the ecologically dominant lineage of coccolithophores**

El Mahdi Bendif^1*^, Ian Probert^2,3^, Francisco Díaz-Rosas ^4,5,6^, Daniela Thomas^4,5,6^, Ger van den Engh^7^, Jeremy R. Young^8^ and Peter von Dassow^4.5.6 *^

^1^ Marine Biological Association of the UK, Plymouth, UK

^2^ Université Pierre et Marie Curie (Paris VI), Roscoff Culture Collection, Station Biologique de Roscoff, Roscoff, France

^3^ Centre National de la Recherche Scientifique, FR2424, Station Biologique de Roscoff, Roscoff, France

^4^ Facultad de Ciencias Biológicas, Pontificia Universidad Católica de Chile, Santiago, Chile

^5^ Instituto Milenio de Oceanografía, Chile

^6^ UMI 3614, Evolutionary Biology and Ecology of Algae, CNRS-UPMC Sorbonne Universités, PUCCh, UACH, Station Biologique de Roscoff, Roscoff, France

^7^ Center for Marine Cytometry, Concrete, WA, USA

^8^ Departments of Earth Sciences, University College London, London, UK

*** Correspondences:**

**Peter von Dassow**

**pvondassow@bio.puc.cl**

**El Mahdi Bendif**

**elmhidi@gmail.com**

**Ian Probert**

**probert@sb-roscoff.fr**

## Supplementary Figures


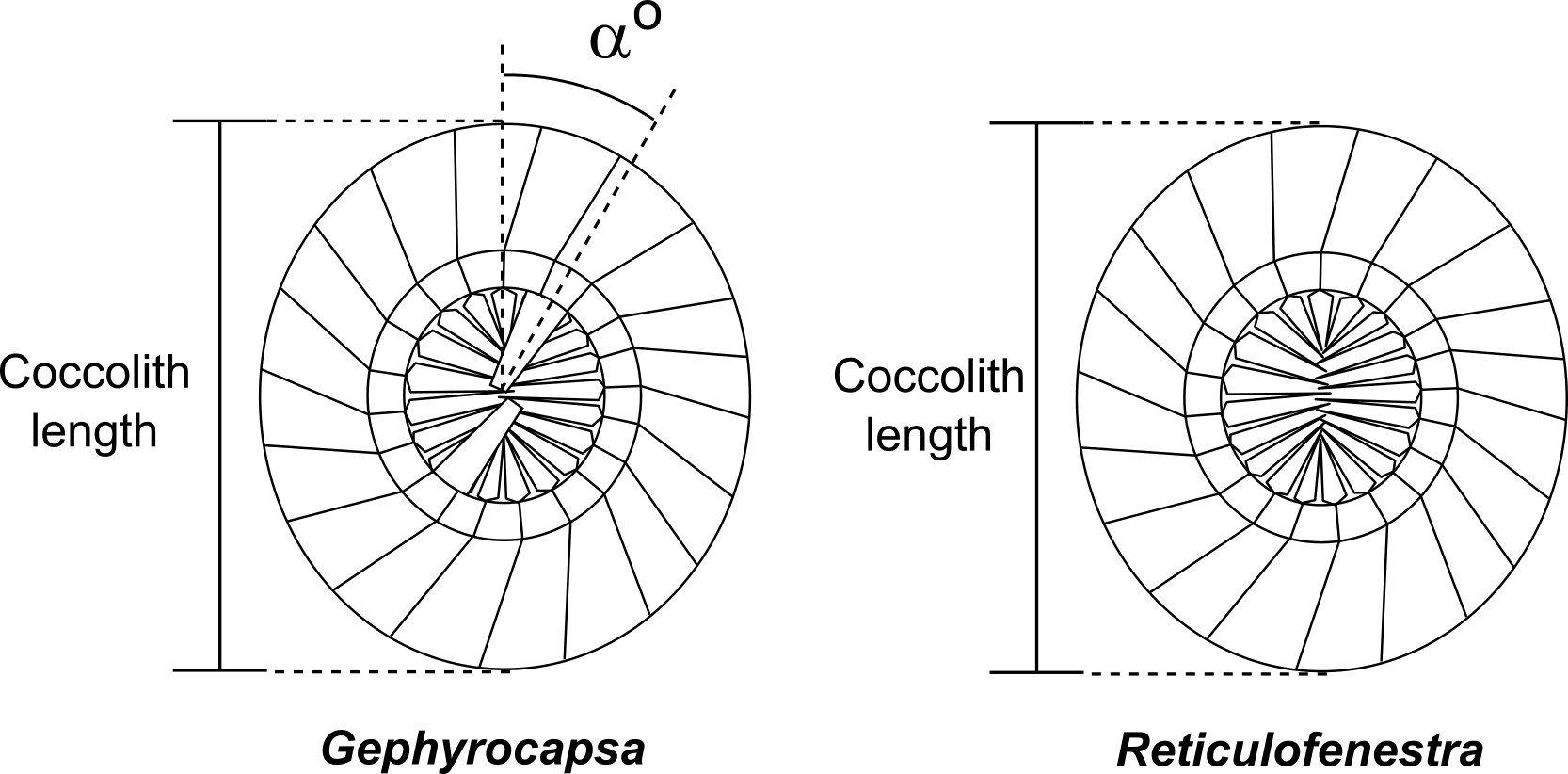


Supplementary figure 1. Morphometric features measured on new isolates.


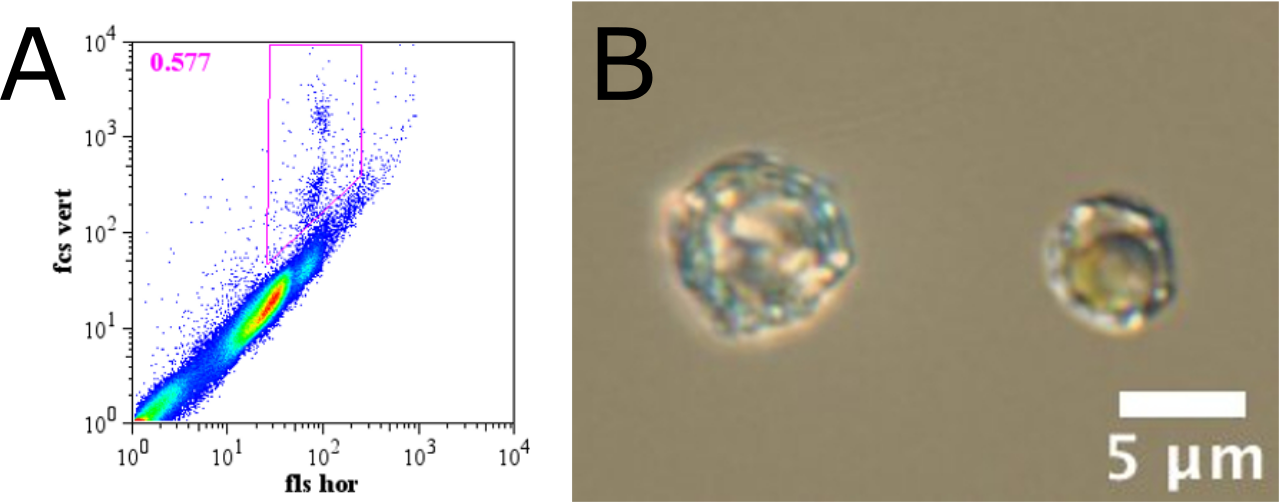


Supplementary figure 2. (A) Flow cytometry plot of sample NBP568 with two populations highlighted in the pink-box; (B) Light Microscopy micrograph of the two sized-type populations.


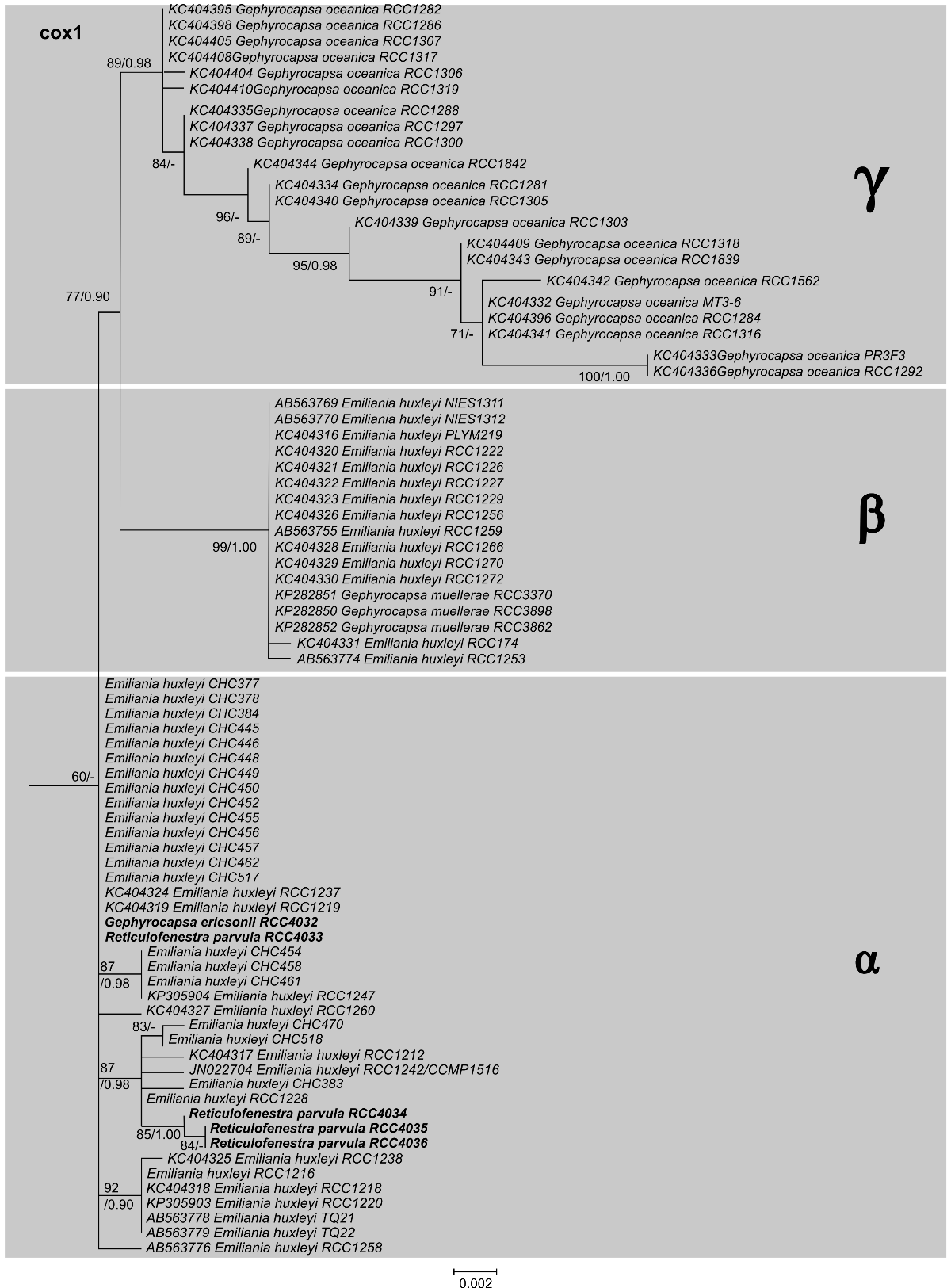


Supplementary figure 3. Molecular phylogenies of the Noëlaerhabdaceae inferred from comparisons of mitochondrial *cox1* sequences. Support values at each node are presented for ML/Bayes analyses. Bootstrap values larger than 50 and posterior probabilities larger than 0.80 are shown. Lesser values are represented by “–”.


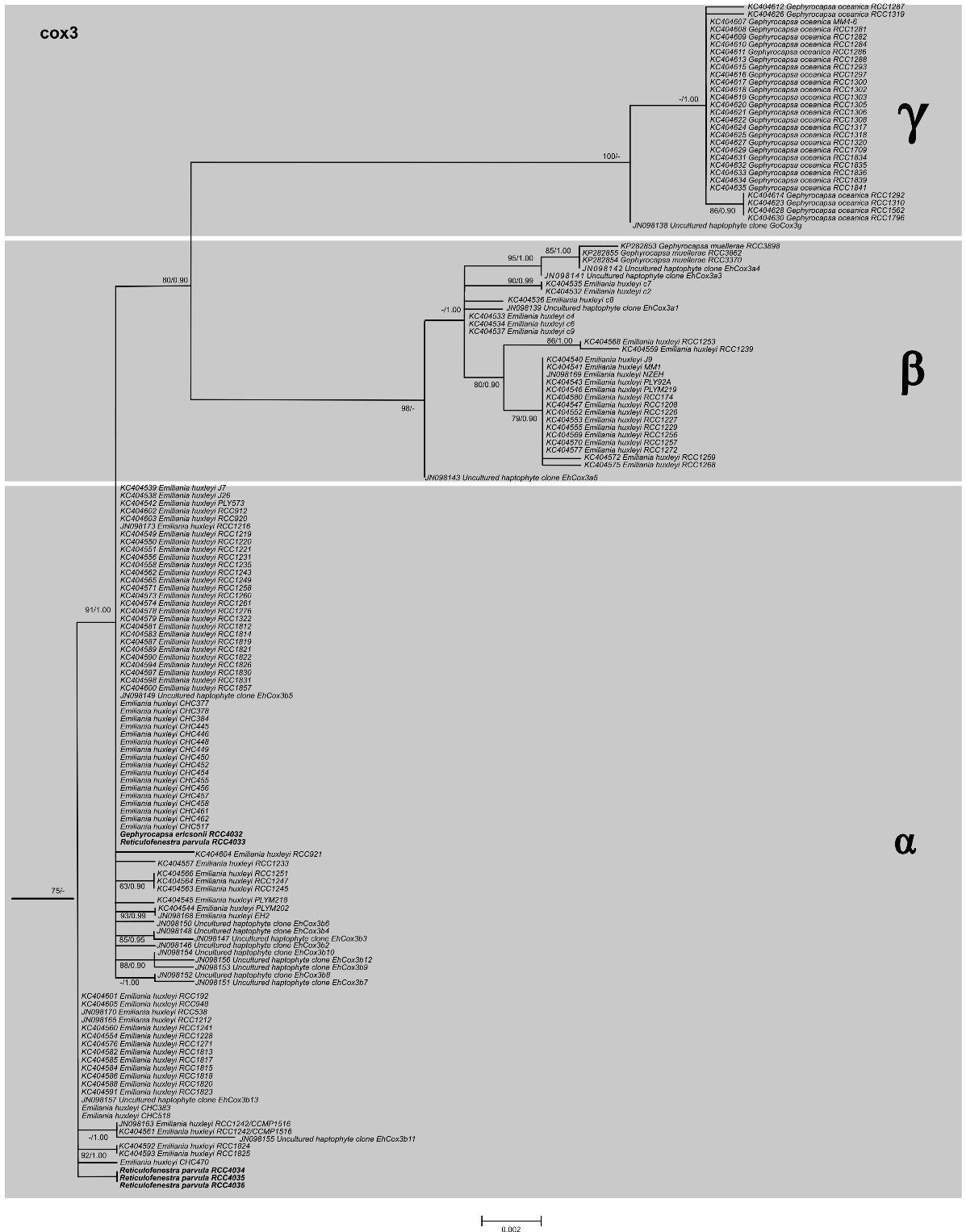


Supplementary figure 4. Molecular phylogenies of the Noëlaerhabdaceae inferred from comparisons of mitochondrial *cox3* sequences. Support values at each node are presented for ML/Bayes analyses. Bootstrap values larger than 50 and posterior probabilities larger than 0.80 are shown. Lesser values are represented by “–”.


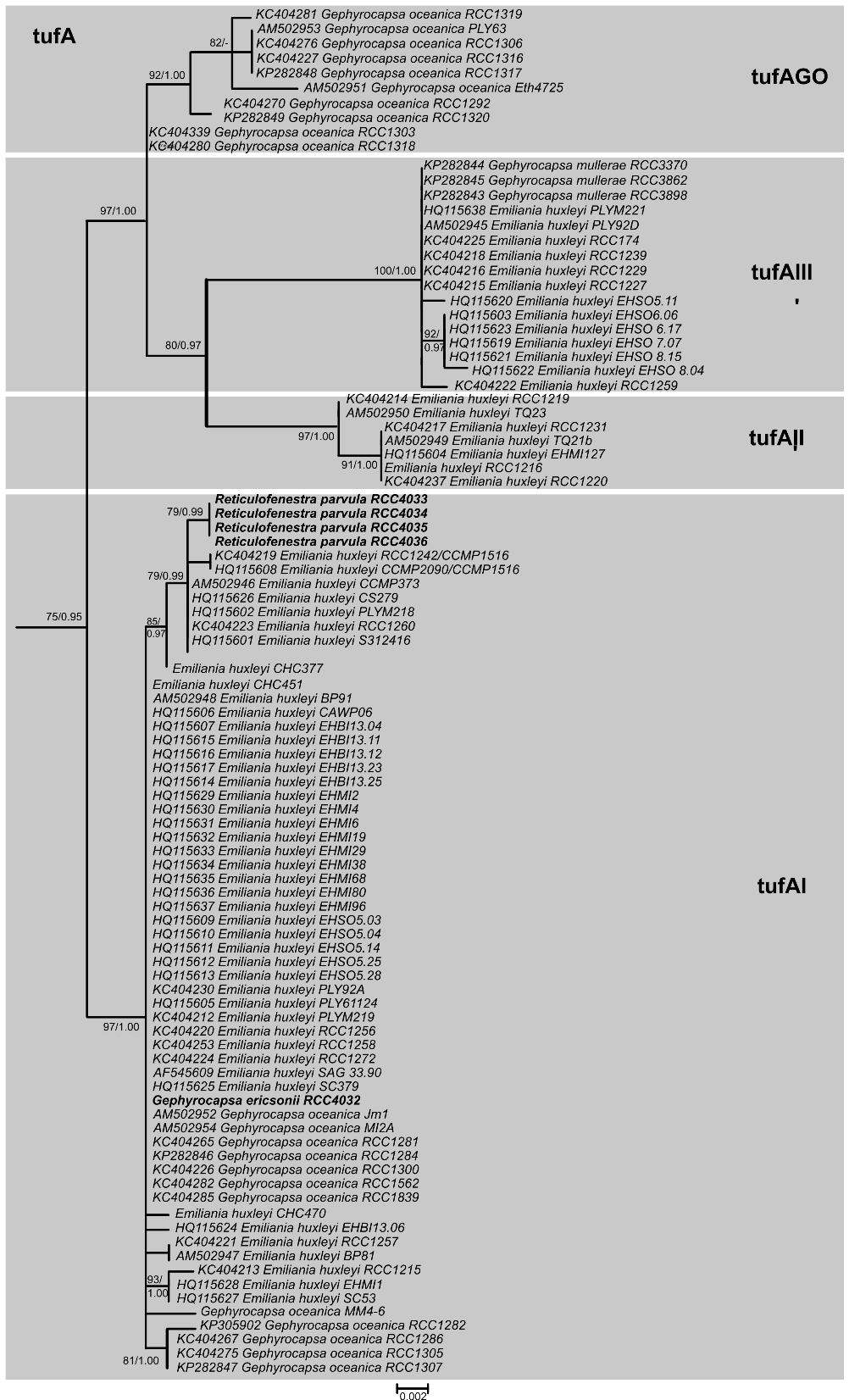


Supplementary figure 5. Molecular phylogenies of the Noëlaerhabdaceae inferred from comparisons of plastidial *tufA* gene sequences. Support values at each node are presented for ML/Bayes analyses. Bootstrap values larger than 50 and posterior probabilities larger than 0.80 are shown. Lesser values are represented by “–”.

**
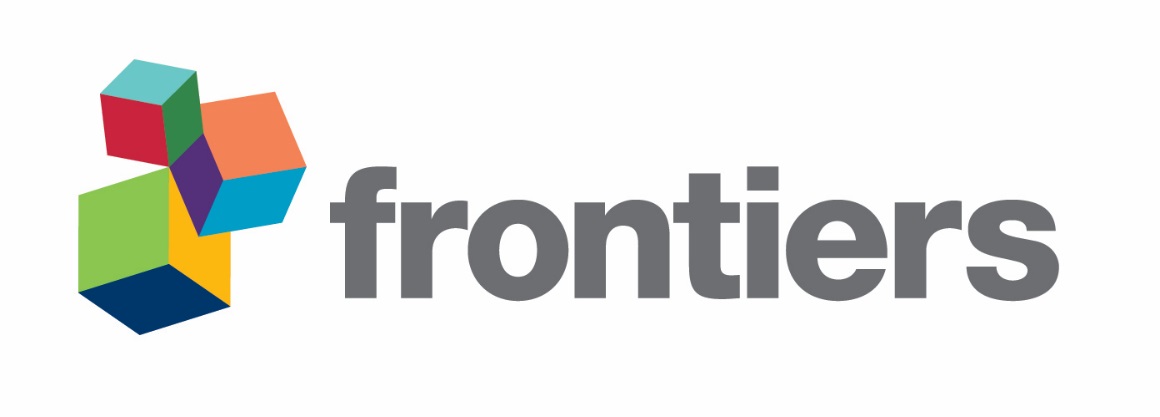
**
